# Facultative methanotrophs – diversity, genetics, molecular ecology and biotechnological potential: a mini-review

**DOI:** 10.1099/mic.0.000977

**Published:** 2020-10-21

**Authors:** Muhammad Farhan Ul Haque, Hui-Juan Xu, J. Colin Murrell, Andrew Crombie

**Affiliations:** ^1^​ School of Biological Sciences, University of the Punjab, Lahore, Pakistan; ^2^​ School of Environmental Sciences, University of East Anglia, Norwich, NR4 7TJ, UK; ^3^​ School of Biological Sciences, University of East Anglia, Norwich, NR4 7TJ, UK; ^†^​ Present address: Joint Institute for Environmental Research & Education, College of Natural Resources and Environment, South China Agricultural University, Guangzhou 510642, PR China; ^‡^​ Present address: School of Environmental Sciences, University of East Anglia, Norwich, NR4 7TJ, UK

**Keywords:** biogeochemical cycling, facultative methanotrophs, Methane, Methane monooxygenase, *Methylocella*, Methylocystis, Methylocapsa

## Abstract

Methane-oxidizing bacteria (methanotrophs) play a vital role in reducing atmospheric methane emissions, and hence mitigating their potent global warming effects. A significant proportion of the methane released is thermogenic natural gas, containing associated short-chain alkanes as well as methane. It was one hundred years following the description of methanotrophs that facultative strains were discovered and validly described. These can use some multi-carbon compounds in addition to methane, often small organic acids, such as acetate, or ethanol, although *Methylocella* strains can also use short-chain alkanes, presumably deriving a competitive advantage from this metabolic versatility. Here, we review the diversity and molecular ecology of facultative methanotrophs. We discuss the genetic potential of the known strains and outline the consequent benefits they may obtain. Finally, we review the biotechnological promise of these fascinating microbes.

## The global methane budget and its significance for climate

**Fig. 1.**
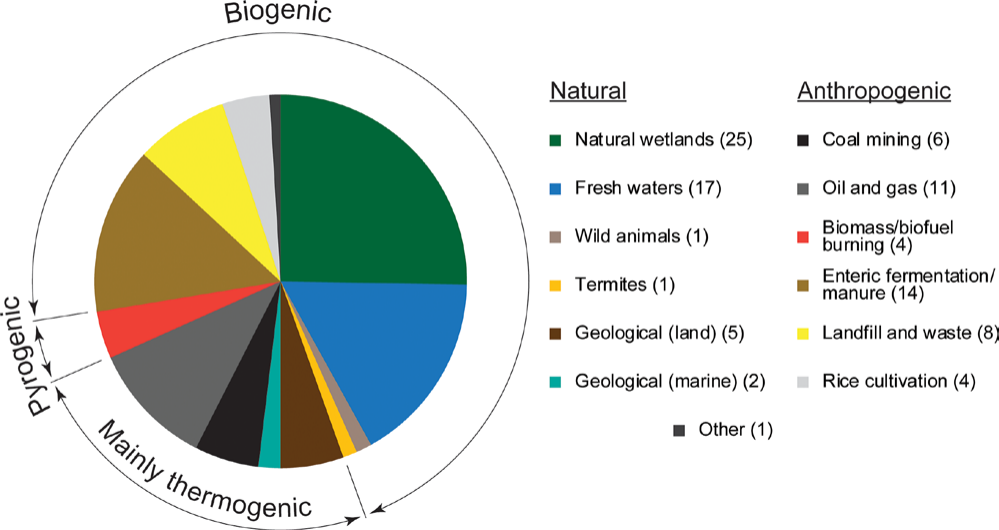
Summary of natural and anthropogenic methane sources to the atmosphere. A proportion of coal bed methane is of biogenic origin [179]. The magnitude of each source as a percentage of the total (736 Tg CH_4_ y^−1^) is shown in parentheses. Data from reference [1].

Methane, the most abundant hydrocarbon in the atmosphere and a potent greenhouse gas, is one of the most significant contributors to climate change. The atmospheric concentration of methane increased to over 1800 ppb by 2012, 2.5 times the pre-industrial value [1]. Moreover, methane is a much more effective greenhouse gas than carbon dioxide, with a global warming potential 28 times that of CO_2_ (per unit mass) over 100 years. The short lifetime in the atmosphere (approximately 9 years [2]) and relatively minor source/sink imbalance suggests that reduction in methane emissions would have rapid and relatively achievable benefits for climate [3], but clearly prediction and mitigation of methane emissions requires a comprehensive understanding of global and regional-scale budgets, and of how the sources and sinks respond to changing conditions. Globally, 540–884 Tg of methane are emitted annually from various natural and anthropogenic sources [1] (Fig. 1). Apart from a minor abiotic (chemical) source [4], methane arises from the biological degradation of organic matter, either by the activity of methanogenic archaea, or by the heat and pressure-mediated breakdown of subterranean organic material over geological timescales, or by the incomplete burning of biomass (termed biogenic, thermogenic or pyrogenic methane, respectively).

Biogenic methane is produced by methanogenic Archaea under anaerobic conditions, mainly in wetlands, landfill sites, rice paddies, the rumen of cattle and the hindgut of termites. Thermogenic methane, the other major source, is of geological origin, produced from the chemical decay of buried sedimentary organic material. Thermogenic methane emissions derive from both anthropogenic fossil fuel extraction and distribution (114–133 Tg y^−1^), and natural sources. The natural sources include macro- and micro-seeps, mud volcanoes, geothermal areas, volcanoes and submarine seeps (33–75 Tg y^−1^) [1]. This thermogenic ‘natural gas’ contains substantial amounts of other climate-active gases, mainly ethane (a photochemical pollutant) and propane (an ozone precursor), 2–4 and 1–2.4 Tg y^−1^ from natural sources, respectively [5, 6]. Visible seepage (macro-seeps) and diffuse but pervasive micro-seepage occurs over a considerable proportion of the Earth’s surface, including much of Northern Europe and Russia, and many regions in the USA, including the Appalachian Basin [7], where the gas contains, in addition to methane, up to 35 vol% ethane and propane [8, 9].

A large proportion (over 50% [10]) of biogenic and thermogenic methane is subsequently consumed in both anoxic and oxic zones by methane-oxidizing microbes (methanotrophs) before its release to the atmosphere. The major sink for atmospheric methane (90 %) is photochemical oxidation by hydroxyl radicals, predominately in the troposphere but also in the stratosphere [11], although soil-dwelling methanotrophs draw down approximately 30 Tg y^−1^ of atmospheric methane [12, 13].

## Microbial growth on methane and short-chain alkanes

Methanotrophs, which are widespread in freshwater, marine and terrestrial environments, are bacteria able to grow on methane as their sole source of carbon and energy and are a subset of methylotrophs, micro-organisms that grow on one-carbon compounds such as methanol and methylated amines. Anaerobic oxidation of methane plays an important role in mediating emissions in anoxic zones, principally in marine but also in freshwater environments [14, 15], but here we consider only the aerobes. All aerobic methanotrophs use a methane monooxygenase (MMO) to oxidize methane to methanol, which is further oxidized to formaldehyde by methanol dehydrogenase (MDH). There are two forms of MMO, a membrane-associated copper-containing enzyme (particulate methane monooxygenase, pMMO) and a cytoplasmic enzyme (soluble methane monooxygenase, sMMO). The sMMOs form one group of a large family of soluble diiron centre monooxygenases (SDIMOs), which bacteria use to grow on a wide range of hydrocarbons and which, based on DNA sequence, gene layout, subunit composition and substrate specificity, can be assigned to one of six major groups [16, 17]. The growth substrates of SDIMO groups 1 and 2 are aromatic compounds or alkenes, group 3 comprises the sMMOs, group 4 contains alkene monooxygenase and groups 5 and 6 contain mainly propane monooxygenases. Nearly all methanotrophs possess the pMMO, and a minority also possess the sMMO. *Methylocella* spp. and two strains from the genera *Methyloferula* and *Methyloceanibacter* do not contain the pMMO and thus rely solely on the sMMO to oxidize methane. In strains that use both enzymes, the expression and activity of these enzymes is controlled by copper (the ‘copper switch’), reviewed by Semrau *et al.* [18].

Microbes growing on other short-chain alkanes (e.g. ethane, propane or butane) have also been characterized. Many of these are Gram-positive Actinobacteria, including *Rhodococcus *, *Nocardioides* and *Mycobacterium* but also Proteobacteria, for example *Pseudomonas* [19, 20]. The initial oxidation of short-chain alkanes is usually catalysed by a monooxygenase, frequently an SDIMO related to the group 3 methane monooxygenases, but which is instead from group 5 or group 6 of the SDIMO family [21], although the butane monooxygenase of *Thauera butanivorans* is more closely related to the sMMO [22]. These microbes are metabolically versatile compared to methanotrophs, and generally grow on a range of multicarbon compounds, but not methane [20].

Methanotrophs from approximately two dozen genera are in cultivation, and taxonomically they fall into the classes Alphaproteobacteria and Gammaproteobacteria, and the phylum Verrucomicrobia. In addition, members of the candidate phylum NC10 possess methane monooxygenase and oxidize methane coupled with oxygenic denitrification in anoxic conditions [15]. Historically, the proteobacterial methanotrophs were subdivided into type I and type II, based on physiological traits, including the arrangement of intracytoplasmic membranes, which also corresponds with their taxonomy (Gammaproteobacteria or Alphaproteobacteria) and assimilation of carbon via the RuMP pathway or the serine cycle, respectively [23]. Subsequently, with the discovery of more strains including methanotrophs that assimilate carbon autotrophically via the Calvin–Benson–Bassham (CBB) cycle [24, 25], these categories had to be adjusted and additional subdivisions were added, making this distinction less clear-cut [26].

## Facultative methanotrophs

**Fig. 2.**
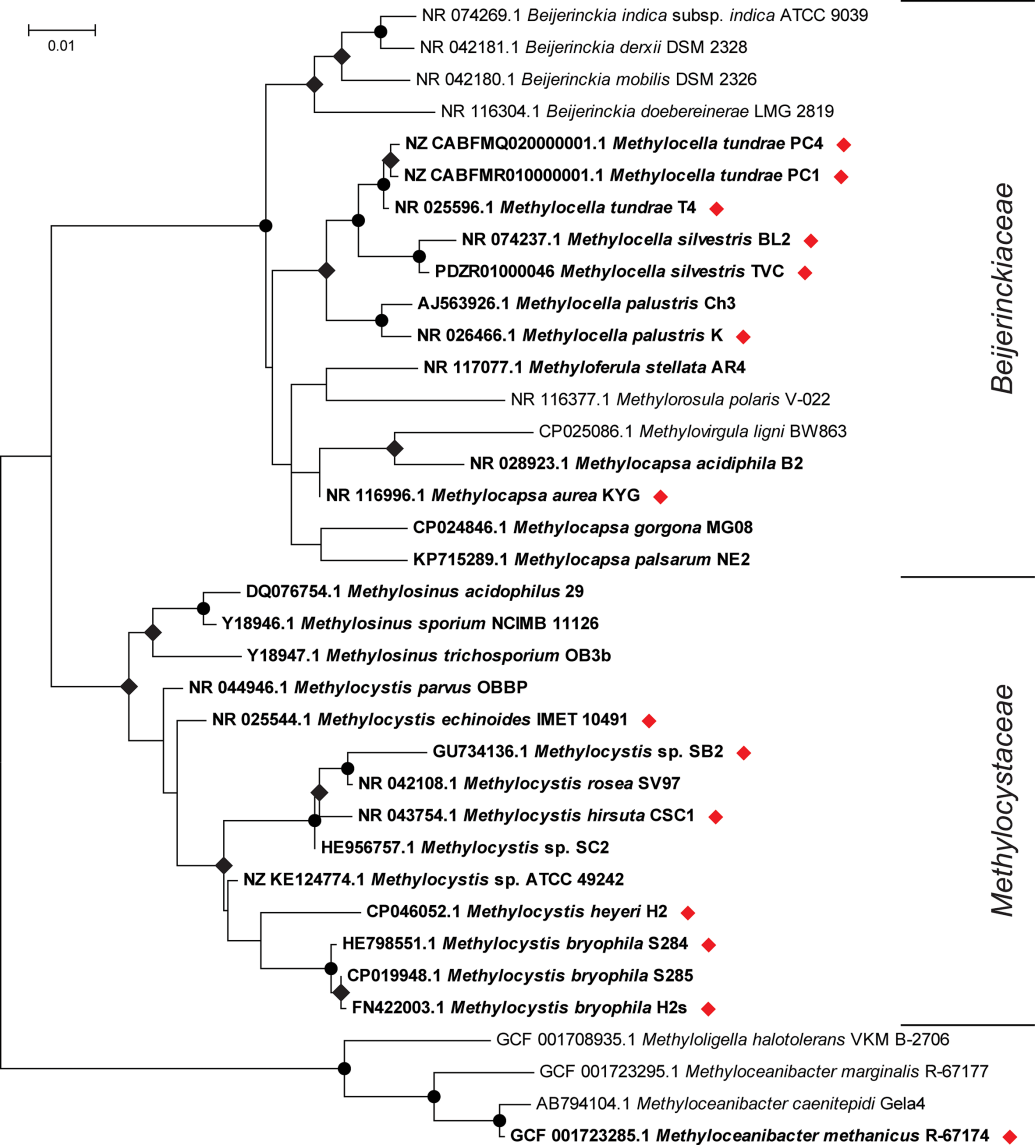
Phylogeny, based on 16S rRNA genes, of alphaproteobacterial methanotrophs (in bold) together with other closely related non-methanotrophic representatives. Facultative strains are identified with red diamonds. The tree was drawn using the maximum-likelihood method in mega7 [183], with bootstrap values (500 replications) greater than 90 or 50 % shown as circles or diamonds, respectively, at the nodes. The tree is drawn to scale and the scale bar indicates substitutions per site. There were a total of 1524 positions in the final dataset.

Although in some cases methanotrophs were shown to assimilate small amounts of carbon from multi-carbon compounds, including carboxylic and amino acids, supplemental to their primary metabolism while growing on methane [27–31], until recently methanotrophy was considered to be an obligate trait; despite several ultimately unconfirmed reports, by the turn of the last century no cultivated examples were known to grow on multi-carbon compounds (containing C–C bonds) as the sole source of carbon and energy, in the absence of methane [32]. In the past two decades, however, using novel media formulations and innovative techniques coupled with improved molecular methods for verification, several facultative methanotrophs have been isolated [33, 34]. These belong to the Alphaproteobacteria; *Methylocystis* (* Methylocystaceae *), *Methyloceanibacter* (Rhizobia *incertae sedis*), or *Methylocapsa* and *Methylocella* (* Beijerinckiaceae *) (Table 1, Fig. 2) [35–39]. *Crenothrix polyspora *, a gammaproteobacterium, was reported to grow on glucose and acetate [40], although no example exists in pure culture. The first of these facultative methanotrophs to be discovered were the *Methylocella* strains, *M. palustris *, *M. silvestris* and *M. tundrae* [41–43]. Although initially described as only growing on C_1_ compounds, they were later shown to use a wide variety of C_2_ – C_6_ multi-carbon compounds including alcohols and organic acids [38, 44, 45] and also, for several of the strains at least, ethane and propane, typical components of thermogenic natural gas. Subsequently, additional facultative strains of *Beijerinckia* and *Methylocystis* were isolated (or identified as facultative), originating from forest, peat or aquifer ecosystems in northern Europe, Russia or the USA (Table 1) as well as a single marine strain, *Methyloceanibacter methanicus* R-67174. In contrast to the *Methylocella* strains, these are much more limited in their substrate utilization, able to use only acetate or ethanol. Their growth rates on these substrates are low compared to on methane, with published rates on acetate or ethanol in the range 7–42 % of the corresponding rate on methane [35–37, 39, 46], in contrast with *Methylocella *, which grows more rapidly on many multi-carbon compounds than on methane [38, 44, 45].

**Table 1.** Facultative methanotrophs

Taxon	Strain	Family	Environment	Location	Multi-C substrates	pH optimum	Ref.
* Methylocystis bryophila *	H2s	* Methylocystaceae *	*Sphagnum* peat	Germany	Acetate	6.0–6.5	[39]
* Methylocystis bryophila *	S284	* Methylocystaceae *	*Sphagnum* peat bog	European N. Russia	Acetate	6.0–6.5	[35]
* Methylocystis echinoides *	IMET 10491	* Methylocystaceae *	Sewage sludge	Germany	Acetate	nr	[180]
* Methylocystis heyeri *	H2	* Methylocystaceae *	*Sphagnum* peat	Germany	Acetate	5.8–6.2	[181]
* Methylocystis hirsuta *	CSC1	* Methylocystaceae *	Uncontaminated aquifer	CA, USA	Acetate	7.0	[182]
* Methylocystis* sp. SB2	SB2	* Methylocystaceae *	Spring bog	MI, USA	Acetate, ethanol	6.8	[37]
* Methylocapsa aurea *	KYG	* Beijerinckiaceae *	Forest soil	Germany	Acetate	6.0–6.2	[36]
* Methylocella silvestris *	BL2	* Beijerinckiaceae *	Forest soil	Germany	Organic acids, alcohols, ethane, propane	5.5	[43]
* Methylocella palustris *	K	* Beijerinckiaceae *	*Sphagnum* peat bog	W.Siberia	Organic acids, alcohols	5.5	[42]
* Methylocella tundrae *	T4	* Beijerinckiaceae *	*Sphagnum* peat	N.Russia	Organic acids, alcohols	5.5–6.0	[41]
* Methylocella silvestris *	TVC	* Beijerinckiaceae *	Tundra soil	N.Canada	Organic acids, ethanol, propane	5.8	[47]
* Methylocella tundrae *	PC1	* Beijerinckiaceae *	Stream water and sediment	NY, USA	Organic acids, alcohols, ethane, propane	5.8	[45]
* Methylocella tundrae *	PC4	* Beijerinckiaceae *	Stream water and sediment	NY, USA	Organic acids, alcohols, ethane, propane	5.8	[45]
* Methyloceanibacter methanicus *	R-67174	Rhizobiales *incertae sedis*	Marine sediment	North Sea	Acetate	7.3	[49]

nr, not reported.

Recent efforts to obtain additional *Methylocella* strains resulted in isolation of *Methylocella silvestris* strain TVC, originating from stream sediment/soil from a permafrost location in N. Canada [47]. Next, the observation that *Methylocella silvestris* could grow on propane and methane concurrently [44], prompted sampling from environments where these gases co-occur, specifically natural gas seeps at sites including streams in northern New York State, where ethane and propane together comprise up to 35 % v/v [9, 48]. Two isolates, *Methylocella tundrae* strains PC1 and PC4, were obtained from these environments [45].

## Methane and alkane monooxygenases of facultative methanotrophs

**Fig. 3.**
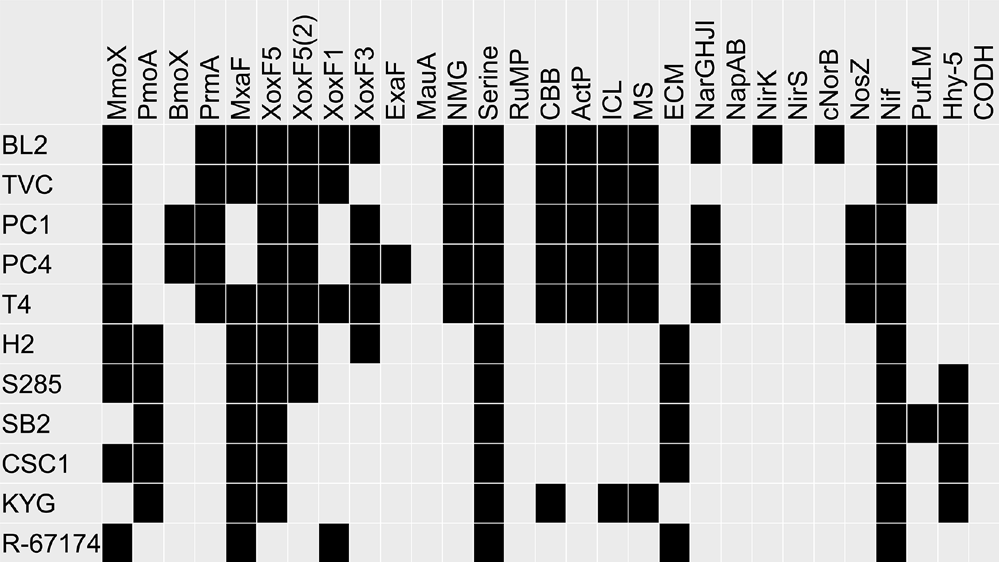
Genetic potential of facultative methanotrophs with determined genome sequences. The presence of a gene or genes encoding a reaction or pathway is shown as black squares. Whole-genome nucleotide sequences were searched with representative protein query sequences using TBLASTN [184]. BL2, *Methylocella silvestris* BL2; TVC, *Methylocella silvestris* TVC; PC1, *Methylocella tundrae* PC1; PC4, *Methylocella tundrae* PC4; T4, *Methylocella tundrae* T4; H2, *Methylocystis heyeri* H2; S285, *Methylocystis bryophila* S285; SB2, *Methylocystis* sp. SB2; CSC1, *Methylocystis hirsuta* CSC1; KYG, *Methylocapsa aurea* KYG; R-67164, *Methyloceanibacter methanicus* R-67174. MmoX, soluble methane monooxygenase; PmoA, particulate methane monooxygenase; BmoX, butane monooxygenase; PrmA, propane monooxygenase; MxaF, Ca-dependent methanol dehydrogenase; XoxF, lanthanide-dependent methanol dehydrogenase; ExaF, lanthanide-dependent ethanol dehydrogenase; MauA, methylamine dehydrogenase; NMG, N*-*methylglutamate pathway; Serine, serine cycle; RuMP, ribulose monophosphate pathway; CBB, Calvin–Benson–Bassham pathway; ActP, acetate-specific permease; ICL, isocitrate lyase; MS, malate synthase; ECM, ethylmalonyl-CoA pathway; NarGHJI, respiratory nitrate reductase; NapAB, periplasmic nitrate reductase; NirK, copper-containing nitrite reductase; NirS, multi-haem nitrite reductase; cNorB, cytochrome *c*-dependent nitric oxide reductase; NosZ, nitrous oxide reductase; Nif, nitrogenase; PufLM, photosynthetic reaction centre; Hhy-5, high-affinity group 5 hydrogenase; CODH, carbon monoxide dehydrogenase.

**Fig. 4.**
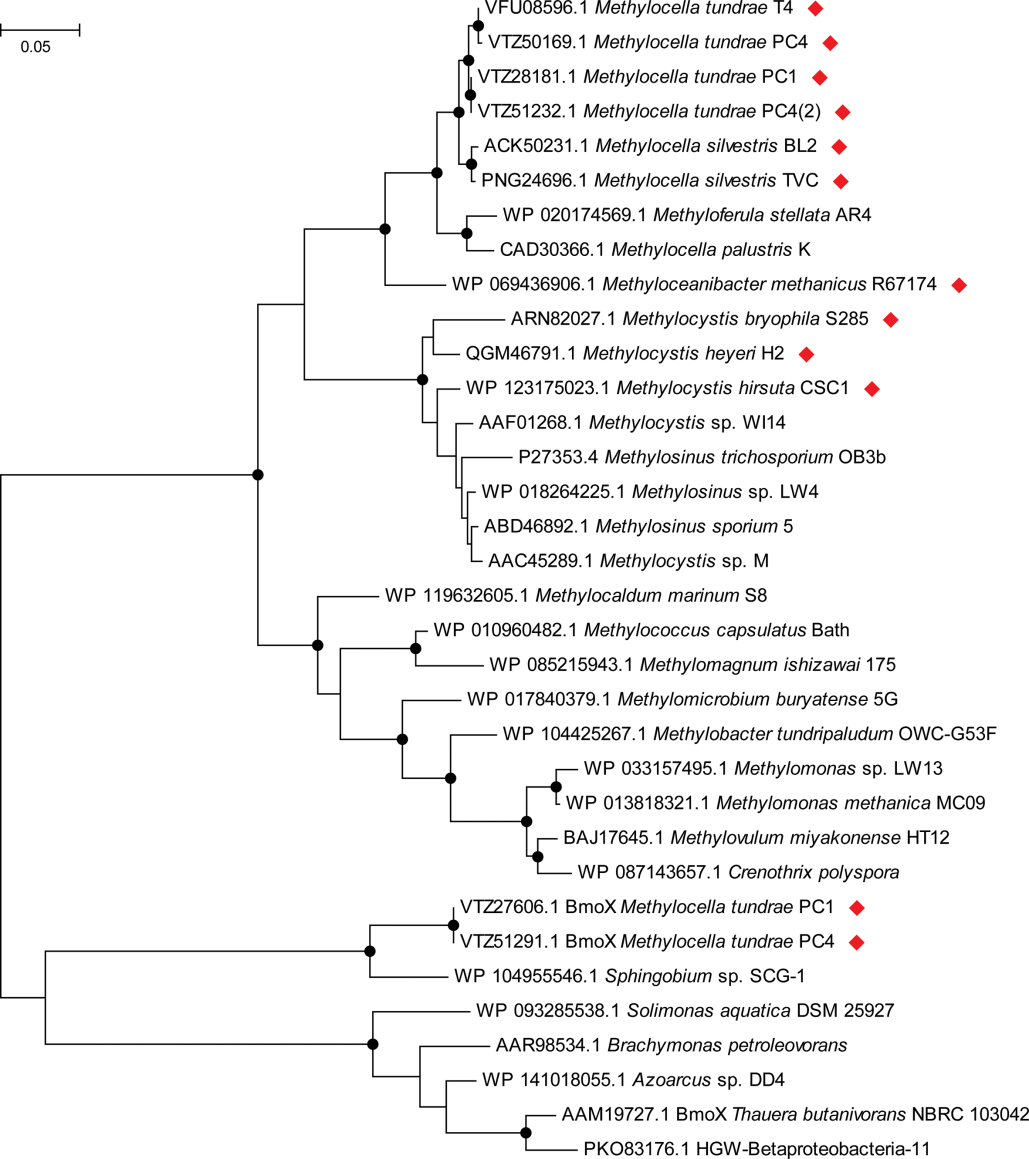
Relationship of the α-subunits of the sMMOs and sMMO-like proteins from facultative methanotrophs (indicated with red diamonds) and other representative strains. The sequences at the bottom of the figure, which form a group with BmoX (butane monooxygenase) of *Thauera butanivorans,* are from non-methanotrophs, except for those from *Methylocella tundrae* strains PC1 and PC4. The tree was drawn using the maximum-likelihood method in mega7 [183], with bootstrap values (500 replications) greater than 75 % shown as solid circles at the nodes. The tree is drawn to scale and the scale bar indicates substitutions per site. There were a total of 540 amino acid residues in the final dataset.

So far, all known *Methylocella* strains differ from almost all other methanotrophs in possessing only the sMMO rather than the copper-dependent membrane-associated pMMO, the only other sMMO-only methanotrophs described being *Methyloferula stellata* AR4 and *Methyloceanibacter methanicus* R-67174. Interestingly, the latter strain is facultative and grows to a low density on acetate, although it is the only methane-utilizing member of this genus, which otherwise contains facultative methylotrophs able to grow on several multi-carbon compounds [49–51]. Of the other facultative methanotroph strains, *Methylocapsa aurea* and *Methylocystis* sp. SB2 contain only the pMMO, whereas the rest contain both forms of MMO (Fig. 3). *M. tundrae* PC4 contains two similar but not identical sMMO operons (96 % nucleotide identity between the operons *mmoXYBZDCRG*, 88–99% amino acid identity between polypeptides). Although many methanotrophs contain multiple copies of the *pmoCAB* operon, additional copies of the sMMO genes are hitherto unknown [52]. *M. tundrae* strains PC1 and PC4 also contain an additional SDIMO gene cluster with identical subunit layout to the sMMO [45]. These MmoX-like sequences form a small subgroup (described as BmoX in Figs 3 and 4), distinct from the MmoX sequences of characterized sMMOs, clustering with a sequence from *Sphingobium* sp. SCG-1, isolated from soil associated with a major natural gas leak [53], and more distantly with BmoX from *Thauera butanivorans *, which grows on butane (but not methane) as the sole source of carbon and energy [54] (Fig. 4). However, neither of these *Methylocella* strains can grow on butane [45], so the role of these genes remains unclear.

**Fig. 5.**
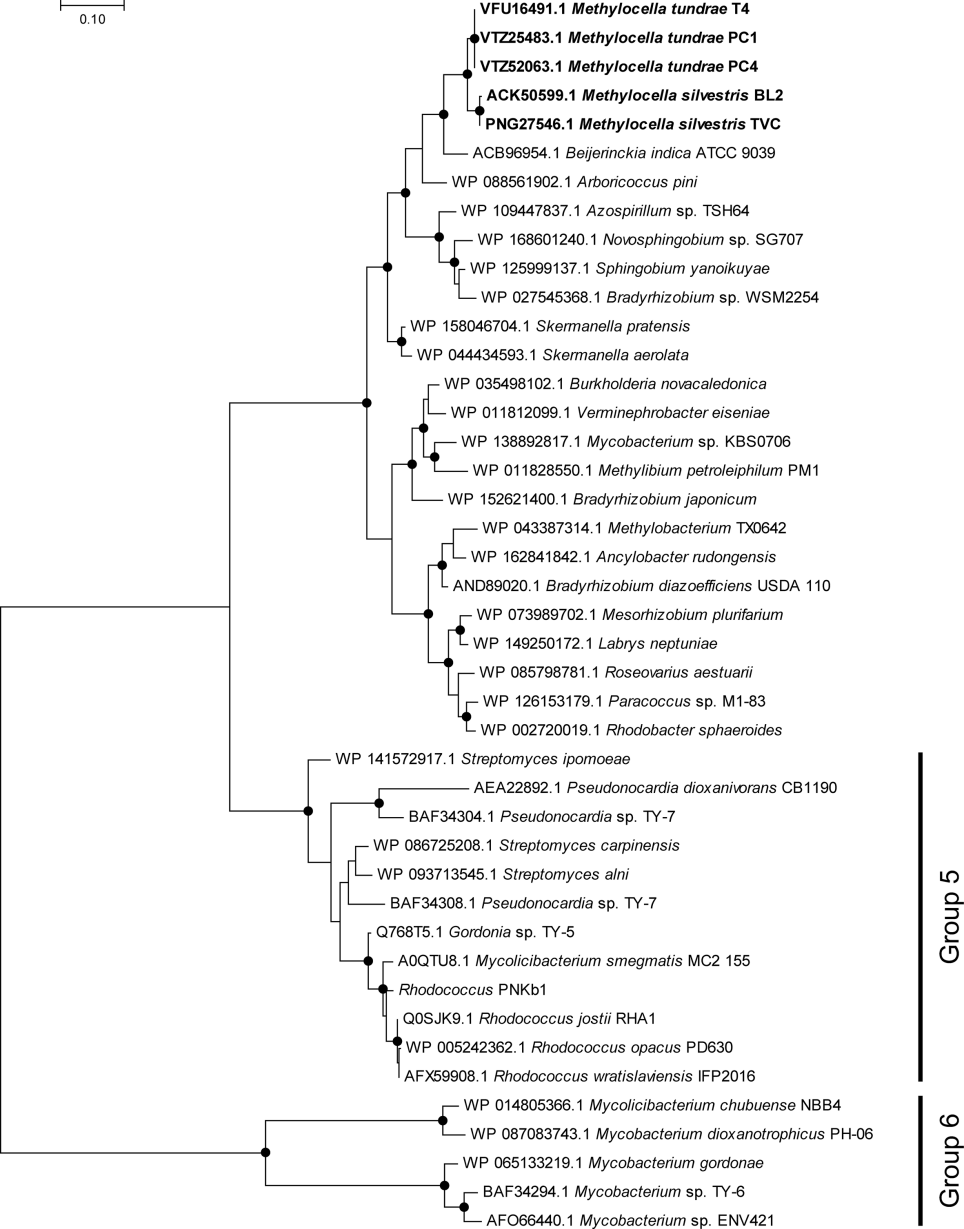
The *Methylocella* PrmA (propane monooxygenase α-subunit) sequences (shown in bold), group with those of diverse strains not known for propane oxidation, distinct from the propanotrophs in SDIMO groups 5 and 6. The tree was drawn using the maximum-likelihood method in mega7 [183], with bootstrap values (500 replications) greater than 75 % shown as solid circles at the nodes. The tree is drawn to scale and the scale bar indicates substitutions per site. There were a total of 440 amino acid residues in the final dataset.

All the *Methylocella* strains contain a propane monooxygenase-like (PrMO) sequence belonging to SDIMO group 5 [17], and several of them have been tested and can grow on propane [44, 45, 47, 55]. The hydroxylase alpha subunits (encoded by *prmA*) form a distinct cluster within group 5, distinct from the group 5 and 6 enzymes of characterized propanotrophs such as *Gordonia* sp. TY-5, *Rhodococcus* sp. RHA1 or *Mycobacterium* sp. TY-6, and instead cluster with sequences from a diverse range of organisms not known for growth on short-chain alkanes (Fig. 5). Interestingly, this *prm* gene cluster, together with genes encoding propionyl-CoA carboxylase and methylmalonyl-CoA mutase and epimerase, involved in the subsequent metabolism of 1-propanol (the product of terminal propane oxidation), are located on a megaplasmid in *M. tundrae* T4, but chromosomally encoded in *M. silvestris* [55, 56]. In *Methylocella silvestris* BL2, while *prmA* was required for growth on propane at high concentrations (20 % v/v), it was not essential for growth at the lower concentrations typically found in the natural environment [44]. However, the propane monooxygenase was implicated in growth on 2-propanol and acetone, as it was in other bacteria [57, 58], suggesting that the environmental role of this enzyme is not restricted to oxidation of short-chain alkanes.

## Alcohol oxidation and one-carbon assimilation

Methanol dehydrogenase (MDH), the second essential enzyme for methane metabolism, catalyses the conversion of methanol to formaldehyde. The classical MDH is a soluble periplasmic pyrroloquinoline quinone (PQQ)-containing enzyme, with an α_2_β_2_ structure, consisting of two large subunits (MxaF) and two small subunits (MxaI) and containing a Ca^2+^ ion at the active site [59]. MDH passes electrons to a specific cytochrome *c*_L_ (MxaG), and the *mxa* operon also encodes several genes responsible for Ca insertion and enzyme maturation, including periplasmic solute-binding protein MxaJ, of unknown function. Relatively recently a homologous MDH (Xox-MDH) dependent on a lanthanide (Ln) rare-earth element, rather than calcium, was identified [60]. Although *xoxF* genes (encoding the lanthanide-dependent subunit) were detected in methanotrophs many years ago, their function was not established until the discovery of the role of lanthanides as co-factors [61]. Many methylotrophs and methanotrophs contain both forms of MDH and recently several studies have shown that lanthanides regulate their relative expression and activity [62–64]. The fact that rare-earth elements are not actually rare, but occur in the Earth’s crust in similar amounts to other metals with biological importance (e.g. copper or zinc [65]), and that Xox genes and enzymes are highly active and abundant, suggests that Xox-MDHs may be at least as important as Mxa-MDHs [66, 67]. The *xox* operons are less complex than *mxa* operons; Xox seems not to require the accessory genes required by the calcium-containing MDH and, in addition, most characterized examples lack the small subunit. Xox-type MDHs can be subdivided into five clades, XoxF1–XoxF5, with additional lanthanide-dependent homologues with non-methanol substrates [68]. Of the Xox-MDHs, clade XoxF5 appears to be the most abundant, except that XoxF4 is present to the exclusion of XoxF5 in the order Methylophilales. The structures of examples from clade 2 and clade 5 have been determined [61, 69–71] and several enzymes have been purified [68]. The Ln-dependent enzymes can be identified by the presence of an Ln-coordinating Asp residue two positions from the catalytic Asp residue (highly conserved in both MxaF and XoxF proteins), which is occupied by Ala in MxaF proteins [68, 71, 72]. Virtually all methanotrophs that contain Mxa-MDH also contain Xox-MDH, often more than one copy and sometimes from different clades [73, 74]. The advantage to a methanotroph of this methanol-oxidizing flexibility is unclear, but it may be important as a response to changing environmental conditions or for controlling symbiotic transfer of metabolites (e.g. methanol) to other members of the microbial community [75, 76].

All the facultative methanotroph strains except for *Methyloceanibacter* (which encodes XoxF1) encode one or two copies of XoxF5 (Fig. 3). The *Methylocella* strains also encode one or both of clade 1 and clade 3 enzymes. All also encode an Mxa-MDH, except for *M. tundrae* strains PC1 and PC4, which contain only Xox-MDHs [45]. These two strains were unable to grow on methanol in the absence of a rare-earth element, in common with a relatively small number of other methylotrophs [61, 77–79], which require these elements for methylotrophic growth. Subsequent to the isolation of these Xox-only *Methylocella* strains, additional methylotrophic (not methanotrophic) Xox-only members of the *Beijerinckiaceae* have been discovered [80]. Some of the facultative methanotrophs can grow on ethane or ethanol and so the identity of the enzyme(s) responsible for ethanol oxidation is of interest. *Methylorubrum extorquens* and *Pseudomonas putida* express lanthanide- and PQQ-dependent dehydrogenases with good ethanol compared to methanol-oxidizing activity, termed ExaF or PedH, respectively [81, 82]. However, in *M. extorquens* both forms of MDH can also efficiently use ethanol as substrate [81, 83], and it required deletion of all three MDHs as well as ExaF to prevent growth on either methanol or ethanol [81]. In contrast, analysis of the *Methylocystis* sp. SB2 transcriptome (grown without added lanthanide) showed that expression of Mxa-MDH was downregulated 50-fold during growth on ethanol in comparison to growth on methane, and expression of an NAD(P)-dependent short-chain dehydrogenase was fivefold upregulated [84] suggesting that the MDH is not primarily responsible for ethanol oxidation under these conditions in this strain. Since, of the facultative methanotrophs, only *Methylocella tundrae* PC4 encodes a PQQ-dependent ethanol dehydrogenase homologous to ExaF/PedH (Fig. 3) and since *Methylocella* spp. can grow on a range of alcohols [44, 45, 47], more research is needed to identify the enzymes responsible.

Methylated amines are present in many soils and aquatic environments [85] and many bacteria have evolved to metabolize these compounds, using one of a number of pathways [86]. *Methylocella silvestris* BL2 can grow on mono-, di- or tri-methylamine using the *N*-methylglutamate pathway [87–89] and the required genes (*gmas* and associated genes) are present in all the *Methylocella* isolates, but not in the other facultative strains, although *Methyloceanibacter methanicus* R-67174 appears to contain remnants of this gene cluster, but did not grow on methylamine [49]. Interestingly, none of the facultative methanotroph strains encode the key enzyme of the alternative proteobacterial pathway, methylamine dehydrogenase (MauAB), suggesting that the *Methylocystis* and *Methylocapsa* strains cannot utilize this source of carbon and, perhaps equally important in oligotrophic habitats, nitrogen, thus emphasizing the metabolic versatility of *Methylocella.*

All of the facultative methanotrophs assimilate carbon using the serine cycle (Fig. 3); none of the isolates possesses genes for the key enzymes of the RuMP cycle, 3-hexulose-6-phosphate synthase or 6-phospho-3-hexuloisomerase. However, the *Methylocella* and *Methylocapsa* strains possess CBB cycle genes, of which the deduced RubisCO large subunits (CbbL) share 88–90% amino acid identity with the form I enzyme from *Bradyrhizobium diazoefficiens* [90] and 92–94% identity with CbbL from closely related *Beijerinckia mobilis *, which was reported to grow autotrophically on methanol [91], raising the possibility that the CBB cycle may contribute to carbon fixation in some facultative strains under as-yet undefined conditions.

## Growth on multi-carbon compounds

As mentioned above, the ability to grow on acetate and/or ethanol (and, in the case of *Methylocella *, a range of other multi-C compounds) in addition to methane is the defining feature of all so-far discovered facultative methanotrophs. However, *Methylocella* grows much better on two-carbon compounds than the other strains and it has been suggested that the inability of obligate methanotrophs to grow on multi-carbon compounds, such as acetate, is due to the lack of appropriate membrane transporter mechanisms [34, 92, 93]. In this context, it is interesting to note that whereas all the facultative strains contain a number of relatively uncharacterized membrane transporters, which possibly allow organic acids to enter the cell, only the *Methylocella* strains possess close homologues of *actP*, an acetate-specific permease (67–71% amino acid identity with ActP characterized in *Escherichia coli* [94] and 68–70% identity with META1p2533 from *Methylorubrum extorquens* AM1 [95, 96]).

When two-carbon compounds enter the cell, their assimilation presents another difficulty, since the TCA cycle oxidizes acetyl-CoA to two molecules of CO_2_, generating energy but not supplying carbon for assimilation. For many years, assimilation of acetate was considered to require the activity of the two enzymes of the glyoxylate shunt, isocitrate lyase (ICL) and malate synthase (MS), which together bypass the decarboxylation reactions of the TCA cycle, forming four-carbon molecules from two acetyl-CoA [97]. During one-carbon growth of serine cycle methylotrophs, the formation of glyoxylate (the substrate of the essential serine cycle enzyme serine-glyoxylate aminotransferase), from acetyl-CoA, can be catalysed by ICL together with non-decarboxylating enzymes of the TCA cycle. However, the lack of ICL genes and activity in many methylotrophs prompted researchers to seek an alternative pathway, which was finally resolved with the discovery of the ethylmalonyl-CoA (EMC) pathway (reviewed by Anthony [98]). Interestingly, whereas the *Methylocystis* and *Methyloceanibacter* strains encode the EMC pathway genes, all the *Methylocella* strains and *Methylocapsa aurea* KYG encode the glyoxylate pathway enzymes (Fig. 3), which are comparatively uncommon in serine cycle methanotrophs. Deletion of ICL or MS in *M. silvestris* BL2 resulted in severe growth defects on C_1_ and C_2_ compounds [99, 100], confirming the operation of the glyoxylate shunt in *Methylocella *.

## Additional metabolic capabilities

Methanotrophs use a variety of survival strategies to persist in conditions of varying substrate availability and under the influence of environmental stress. Where resources are scarce, these strategies may include energy supplementation by oxidation of other soil or atmospheric trace gases, or by mechanisms such as phototrophy [101–104]. Utilization of hydrogen by methanotrophs has previously been observed, together with the ability to use this energy source to provide the reducing power required for methane oxidation [105, 106]. For example, *Methylocystis* sp. SC2, grown in batch culture, was able to oxidize comparatively high concentrations of supplied hydrogen under moderately limiting methane and oxygen concentrations, using a low affinity group 1d hydrogenase (also encoded by *Methylocystis* strains H2 and 285), achieving increased biomass yield from methane and depleting hydrogen to below the limits of detection [107]. *Methylocystis* strains 285 and SB2 and *Methylocapsa aurea* KYG encode a high-affinity group 5 NiFe uptake hydrogenase [108], similar to that used by other methanotrophs such as *Verrucomicrobia* to grow on low concentrations of hydrogen [101, 109], potentially allowing these strains to obtain energy from hydrogen in the atmosphere (<1 ppmv) under oligotrophic conditions [110]. *Methylocella silvestris* strains BL2 and TVC and *Methylocapsa aurea* KYG encode a group 2a enzyme, perhaps involved in recycling hydrogen produced as a bi-product of nitrogen fixation, although an enzyme from the same group can oxidize atmospheric hydrogen in *Mycolicobacterium smegmatis* (which also expresses a group 5 enzyme) [111]. Interestingly, the *Methylocella tundrae* strains appear to lack hydrogenases, despite possessing nitrogen fixation genes, suggesting that they are unable to use exogenous or internally produced hydrogen. Moreover, none of the facultative methanotrophs encode a carbon monoxide dehydrogenase, an enzyme used by many soil bacteria to enhance survival in oligotrophic conditions [112].

Aerobic anoxygenic phototrophs are abundant in aquatic ecosystems where the additional energy derived from light may give them a competitive advantage [113–115]. Some phototrophs are methylotrophs, and it is interesting to note that the *Methylocella silvestris* and *Methylocystis* sp. SB2 genomes contain the required *pufLM* and bacteriochlorophyll biosynthesis genes [116, 117] (Fig. 3), although the significance of this is currently unknown.

Where methane is abundant and oxygen may be limiting, some methanotrophs thrive at low-oxygen tensions. At the extreme, *Candidatus* Methylomirabilis oxyfera uses nitric oxide dismutation to generate its own molecular oxygen for use by methane monooxygenase, while other methanotrophs economize on oxygen consumption by denitrification [118–121], iron reduction [122, 123], or by fermentation [124, 125], maintaining a supply of molecular oxygen for methane activation. While the denitrification enzymes are also important for detoxification [126], the use of nitrogen compounds as electron acceptor may be significant in methane oxidation [127]. Of the facultative methanotrophs, only the *Methylocella* strains encode enzymes of the denitrification pathway, with *M. silvestris* BL2 containing all genes required for denitrification as far as nitrous oxide, but lacking *nosZ*, encoding the enzyme catalysing the final reduction step to dinitrogen. This gene is present in *Methylocella tundrae* T4, together with a copy of *norB*, (nitric oxide reductase) although this latter gene contains an internal stop codon, suggesting that it is not functional in *M. tundrae* T4. In common with many methanotrophs, the facultative isolates all contain the genetic requirements for nitrogen fixation (Fig. 3).

## Environmental occurrence and ecology

Methanotrophs from the *Methylocystaceae* (* Methylocystis* and *Methylosinus *) and *Beijerinckiaceae* (* Methylocapsa* and *Methylocella *) have mostly been isolated from wetlands and forest soils in the Northern hemisphere [128] and in these and similar environments they are frequently among the most abundant methanotrophs [129–136]. *Methylocystis* and, to a lesser extent *Methylocella *, are also frequently found in rice paddies [137–139], another major source of methane to the atmosphere. In fact, *Methylocella-*related 16S rRNA gene sequences have been found in many diverse environments, ranging from moderately acidic to alkaline (summarized by Rahman *et. al.* [140]), although we should bear in mind that whereas all members of the *Methylocystaceae* (and of the gammaproteobacterial methanotroph families) are methanotrophs, the same is not true for the *Methylocella* family, *Beijerinckiaceae *, which contains methanotrophs, methylotrophs and heterotrophs, nor for the genus *Methyloceanibacter *, which contains non-methane-oxidizing facultative methylotrophs [51, 92]. The difficulty of distinguishing methanotrophs of the *Beijerinckiaceae* from heterotrophic members of this family is compounded since, before the discovery of *Methylocella *, the pMMO was thought to be diagnostic for all methanotrophs and so gene probes were developed using this gene. These have been extensively used to characterize methanotroph communities [128] but cannot detect *Methylocella* or *Methyloceanibacter *. Therefore, the identification of *Methylocella-*like DNA sequences in environmental DNA does not prove methanotrophy, and further confirmation is required. This can be obtained by methods which identify the community, active in response to methane, such as transcriptomics, proteomics or DNA-stable isotope probing. These methods have identified active *Methylocella* in methane-exposed material from, for example, peatlands, forest soil, landfill cover soil, alkaline coal mine, warm springs, acidic aquifers and natural gas seeps [45, 131, 141–147]. However, a consequence of the frequent reliance on *pmoA* gene probes is that *Methylocella* and other sMMO-only methanotrophs must frequently have been (and still are) overlooked in cultivation-independent studies.

Multi-carbon compounds, such as aliphatic, cyclic and aromatic organic acids including acetate, are frequently detectable in the oxic soil horizons and surface sediments which comprise the habitats of methanotrophs, sometimes reaching millimolar concentrations [148–150]. The impact of alternative carbon sources on methanotrophic activity is not clear; some reports have suggested that organic acids inhibit methane oxidation [151–153] while others showed methane oxidation still occurring in the presence of alternative carbon sources [39, 154, 155], or that acetate stimulated the activity of methanotrophs [156–158]. It was suggested that use of acetate by facultative methanotrophs might be a survival strategy when the supply of methane is intermittent, and reports have shown that transcription of the pMMO genes continues under these conditions [39, 153–155]. In *Methylocella silvestris *, acetate repressed sMMO gene transcription, at least at the concentration tested (5 mM) [159, 160], although this was not the case for propane [44]. Recently, with the availability of additional genome sequences, Farhan Ul Haque *et al.* [161] designed improved *Methylocella *-specific *mmoX* primers, which could be used to quantify *Methylocella *-like sMMO gene sequences in environmental samples. When environments exposed to short-chain-alkane-containing natural gas were examined, *Methylocella* were found to be extraordinarily abundant and active, in some cases making up 85 % of the methanotroph community, or 12 % of the total bacterial population [45, 161]. In these environments *Methylocella* may have benefited from the additional ethane and propane, and/or obligate methanotrophs might have been inhibited by these gases or by the products of their co-oxidation [162].

## Biotechnological potential of facultative methanotrophs

Methanotrophs, which are ubiquitous, have potential for bioremediation of polluted sites. For example, it was shown that introduction of methane enhanced aerobic degradation of halogenated hydrocarbons (reviewed by Semrau [163]). Both forms of the MMO can transform these halogenated compounds, although the data suggested that despite its slower degradation rate, the pMMO was ultimately the more effective system. This being the case, the use of facultative *Methylocystis* strains is attractive. In these strains, the pMMO is expressed in the presence of acetate or ethanol [39, 154], which could be used to provide the reductant required by the MMO. This would both be easier to introduce into polluted sites than methane and would also avoid competition for binding to the monooxygenase. As another example of bioremediation, *Methylocella *, was among bacteria associated with degradation of plastics in landfill lysimeters [164, 165].

Obligate methanotrophs such as *Methylococcus capsulatus,* which can grow relatively quickly and to high cell densities, have been exploited for production of single-cell protein [166]. While *Methylocella *, which grows more slowly, may not offer the same potential for the production of low value, bulk chemicals, it can still be grown to high cell densities in fermenter culture [44, 159] and due to its metabolic versatility it should be examined further in this respect. Large-scale production of methanol from methane is an attractive proposition and promising results have been obtained in several studies [167]. For example, co-cultures of *Methylomonas methanica* with *Methylocella tundrae *, immobilized in silica gel, were fed with simulated biogas. Interestingly, methanol production was enhanced (nearly 100 %) by addition of hydrogen, achieving approximately 0.32 g l^−1^ and 66 % conversion efficiency [168]. An obstacle to the use of the MMO to produce methanol is the requirement to supply a costly electron donor (formate) to enable methane oxidation, but facultative methanotrophs may offer an attractive solution since they can instead use compounds, for example acetate, frequently present in the waste stream [169].

The biocatalytic potential of *Methylocella* spp. has not so far been exploited, despite their metabolic versatility when compared to obligate methanotrophs. The sMMO has long been regarded as a highly versatile biocatalyst, catalysing the oxidation of a wide range of alkanes, alkenes and even aromatic compounds as large as naphthalene [163, 166, 170]. It is possible to use whole cells of methanotrophs such as *Methylococcus capsulatus* to produce chemicals such as propylene oxide (from propylene), although the toxic nature of this product requires a recycling system to regenerate the whole-cell biocatalyst [171, 172]. If *Methylocella* is less susceptible to the toxic effects of propylene epoxide, it could offer an advantage over *M. capsulatus* for production of this chemical since it could be supplied with alternative energy sources to drive the oxidation of propylene by sMMO. In addition to the metabolic versatility of *Methylocella *, these strains have an advantage over obligate methanotrophs since expression of the sMMO is not repressed by copper [173]. The prospect of using the sMMO as a biocatalyst while using a multi-carbon compound such as succinate or acetate, as carbon and energy source, suggests that *Methylocella* might be useful as a cell platform for production of high value commodities, for example chiral alcohols and epoxides.

## Final conclusions

It was nearly one hundred years after the first description of a methanotroph [174] before facultative methanotrophs were isolated, which highlights the difficulty of identifying this trait in environmental samples [33] but suggests that more facultative strains await discovery. As mentioned above, phylogeny alone cannot identify facultative methanotrophs from any of the currently identified genera, and it is not easy to devise experiments to identify facultative methanotrophs in the environment with methods used, to good effect, for obligate strains, for example using labelled substrates. Where identifiable genetic markers exist for specific metabolic traits, this can be used to screen (meta)genomic libraries derived from labelled nucleic acids (for example in DNA- or RNA-SIP or single-cell labelling experiments [175–177]), followed by targeted isolation techniques [45], but such traits may not always be easy to identify in genomes. This underlines the crucial importance of isolating and characterizing strains in the laboratory, now possible using several innovative techniques [178]. Facultative methanotrophs are clearly competitive in several environments, and research effort should be directed towards describing their behaviour in synthetic and natural communities and identifying the environmental conditions in which their role is important to the wider methane-oxidizing community. For example, is their facultative ability important in competition with obligate methanotrophs in methane-rich environments and how does oxygen availability affect this? What are the conditions where a denitrifying capability is important, and when is the potential to extract energy from alternative sources useful? In biotechnology and bioremediation their unique metabolic potential also justifies investigation.
